# Patient-Clinician Decision Making for Stable Angina: The Role of Health Literacy

**DOI:** 10.5334/egems.306

**Published:** 2019-08-09

**Authors:** Samuel T. Savitz, Claudia C. Dobler, Nilay D. Shah, Antonia V. Bennett, Stacy Cooper Bailey, Stacie B. Dusetzina, W. Schuyler Jones, Sally C. Stearns, Victor M. Montori

**Affiliations:** 1 Kaiser Permanente Division of Research, US; 2 Mayo Clinic, US; 3 University of North Carolina at Chapel Hill, US; 4 Northwestern University, US; 5 Vanderbilt University Medical Center, US; 6 Duke University Medical Center, US

**Keywords:** health literacy, shared decision making, patient-centered care, health services research, stable angina

## Abstract

**Background:**

Stable angina patients have difficulty understanding the tradeoffs between treatment alternatives. In this analysis, we assessed treatment planning conversations for stable angina to determine whether inadequate health literacy acts as a barrier to communication that may partially explain this difficulty.

**Methods:**

We conducted a descriptive analysis of patient questionnaire data from the PCI Choice Trial. The main outcomes were the responses to the Decisional Conflict Scale and the proportion of correct responses to knowledge questions about stable angina. We also conducted a qualitative analysis on recordings of patient-clinician discussions about treatment planning. The recordings were coded with the OPTION12 instrument for shared decision-making. Two analysts independently assessed the number and types of patient questions and expressions of preferences.

**Results:**

Patient engagement did not differ by health literacy level and was generally low for all patients with respect to OPTION12 scores and the number of questions related to clinical aspects of treatment. Patients with inadequate health literacy had significantly higher decisional conflict. However, the proportion of knowledge questions answered correctly did not differ significantly by health literacy level.

**Conclusions:**

Patients with inadequate health literacy had greater decisional conflict but no difference in knowledge compared to patients with adequate health literacy. Inadequate health literacy may act as a barrier to communication, but gaps were found in patient engagement and knowledge for patients of all health literacy levels. The recorded patient-clinician encounters and the health literacy measure were valuable resources for conducting research on care delivery.

## Introduction

Shared decision making (SDM) for medical decisions has become a prominent goal for the U.S. health care system and has been endorsed as the ideal for patient-clinician interaction by the National Academy of Medicine [1,2]. SDM is especially relevant when the ratio of risks to benefits for clinical outcomes are similar and the optimal course of treatment may depend on how patients weigh the risk of complications or side effects, costs of treatment, and the disruption to their day-to-day life caused by receiving treatment.

The treatment of stable angina is a promising area for evaluating the impact of health literacy on patient-clinician communication during treatment decisions. Treatment partially depends on patient preferences [3], so patients’ ability to understand the tradeoffs between treatment options may influence the care they receive. The most common treatments for stable angina are: medication only (treatment with prescription medications), percutaneous coronary intervention (PCI) with medication, and coronary artery bypass grafting (CABG) surgery with medication. The alternatives vary in terms of cost, how long it takes to receive symptom relief, and the type and risk of complications. Medication only is the least expensive alternative and has comparable long-term symptom relief relative to PCI [3,4]. However, a greater share of patients may experience short-term symptom relief from PCI than medication only [4]. PCI is more costly [5] and more invasive than medication only and requires adherence to dual antiplatelet therapy to prevent stent thrombosis [6]. CABG results in more complete symptom reduction than the other alternatives [7,8,9]. But CABG is also the most invasive (with time needed for surgical recovery) [10], most costly [11], and riskiest alternative (with a small risk of death or other serious complications) [12].

In this study, we examined patient-clinician discussions during treatment planning conversations for stable angina. We conducted a secondary analysis of data from the PCI Choice Trial, which was a randomized controlled trial that evaluated the impact of a conversation aid to facilitate shared decision making for stable angina. The PCI Choice Trial found that the conversation aid improved patient knowledge about the comparative effectiveness of treatment alternatives, but did not improve SDM [13]. The goal of the current study was to evaluate the role of health literacy in patient-provider communication regarding treatment planning for stable angina. The first objective was to evaluate patient engagement and SDM to better understand how clinicians communicated with patients and assess whether communication differed by health literacy level. The second objective was to assess whether health literacy was associated with two outcomes from communication during the encounter: patient decisional conflict and knowledge about the comparative effectiveness of treatment alternatives. We hypothesized that patients with inadequate health literacy would have lower patient engagement, less SDM, greater decisional conflict, and lower knowledge than patients with adequate health literacy.

## Methods

### Data Source

We used data from the PCI Choice study, which was a randomized controlled trial to evaluate a conversation aid (PCI Choice) for treatment of stable angina [dataset] [13,14]. The trial enrolled 124 patients who received care at the general cardiology clinic or cardiac catheterization lab at the Mayo Clinic in Rochester, MN. Patients had to be eligible to receive either percutaneous coronary intervention or medication only. About half of the patients received the conversation aid (N = 65) and half received usual care (N = 59). The study collected the following data:

- Patient questionnaires administered before, immediately after, and three months after the encounter. The questionnaires assessed demographics (before encounter), health literacy (before encounter), preferences for role in decision making (before encounter), patient knowledge (immediately after and three months after encounter), and decisional conflict (immediately after encounter).
- Treatments the patient received up to a year following the encounter were abstracted from the medical records.
- Video and audio recordings of patient-clinician interactions. The recordings were only collected for a subset of patients (N = 54) who consented. The level of SDM was assessed by reviewers evaluating the recorded conversations.

Additional details about the trial are available in previous publications [13,15].

### Sample Selection

We illustrate the sample selection process in Appendix Figure 1. We present the full sample and the recording sample separately because some measures were only available for the recording sample. We excluded patients who had missing values for the health literacy items and limited the recording sample to patients who had their encounter recorded. The final samples were 118 for the full sample and 53 for the recording sample.

### Health Literacy Measure

We assessed health literacy using a validated screening question: “How confident are you filling out health care forms by yourself?” [16,17] We defined patients as having ‘inadequate’ health literacy if they answered “Somewhat,” A little bit,” or “Not at all.” [16,17] Otherwise, we classified patients as having ‘adequate’ health literacy. Previous research has demonstrated that this screening question has good sensitivity and specificity for identifying inadequate health literacy relative to validated instruments including the S-TOFHLA and REALM [17]. More information on the health literacy measure appears in Appendix Table 1.

**Table 1:** Descriptive Statistics for Full Sample and Recording Sample by Health Literacy Category.

		Full Sample (N = 118)		Recording Sample (N = 53)	

		Adequate Health Literacy (N = 96)	Inadequate Health Literacy (N = 22)	Adequate Health Literacy (N = 43)	Inadequate Health Literacy (N = 10)

Variable	Units	Mean (SD)	Mean (SD)	Mean (SD)	Mean (SD)

Age at Visit	Years	68.1 (10.3)	71.5 (11.2)	68.2 (10.3)	73.0 (10.4)
Discussion Length	Seconds			824.7 (482.1)	1191.0 (902.6)
**Variable**	**Value**	**Freq (Percent)**	**Freq (Percent)**	**Freq (Percent)**	**Freq (Percent)**

Patient Gender	Female	28 (29%)	4 (18%)	10 (23%)	2 (20%)
	Male	68 (71%)	18 (82%)	33 (77%)	8 (80%)
Educational Attainment	High school or less	19 (20%)	16 (73%)	8 (19%)	8 (80%)
	Some college or assc. degree	38 (41%)	5 (23%)	17 (40%)	2 (20%)
	College grad. or grad./prof. degree	36 (39%)	1 (5%)	17 (40%)	0 (0%)
Cardiac Procedure Received	PCI	24 (25%)	9 (41%)	16 (37%)	4 (40%)
	CABG	7 (7%)	2 (9%)	2 (5%)	0 (0%)
	Neither PCI nor CABG	65 (68%)	11 (50%)	27 (63%)	6 (60%)
Patient Insurance Status	Private	29 (30%)	7 (33%)	13 (30%)	2 (20%)
	Medicare	47 (49%)	12 (57%)	24 (56%)	8 (80%)
	Medicaid	2 (2%)	2 (10%)	1 (2%)	0 (0%)
	Other	18 (19%)	0 (0%)	5 (12%)	0 (0%)
Caregiver or Family Present	No	NA	NA	11 (26%)	1 (10%)
	Yes	NA	NA	32 (74%)	9 (90%)
Treatment Arm	Usual Care	47 (49%)	8 (36%)	18 (42%)	3 (30%)
	Conversation aide	49 (51%)	14 (64%)	25 (58%)	7 (70%)
Clinician Type	Catheterization Nurse	44 (47%)	12 (55%)	22 (52%)	4 (40%)
	Interventionalist MD or PA/NP	28 (30%)	7 (32%)	14 (33%)	3 (30%)
	Non-Interventionalist	21 (23%)	3 (14%)	6 (14%)	3 (30%)
Patient Preferences for Role in Decision Making*	1 (Patient makes decision)	3 (3%)	0 (0%)	0 (0%)	0 (0%)
	2 (Patient makes decision after considering clinician’s opinion)	28 (30%)	4 (19%)	9 (23%)	3 (33%)
	3 (Shared decision)	48 (52%)	11 (52%)	23 (58%)	3 (33%)
	4 (Clinician makes decision after considering patient’s opinion)	14 (15%)	4 (19%)	8 (20%)	1 (11%)
	5 (Clinician makes decision)	0 (0%)	2 (10%)	0 (0%)	2 (22%)

*Note*: SD stands for standard deviation, CABG stands for coronary artery bypass grafting, PCI stands for percutaneous coronary intervention. Categories may not sum to total numbers presented in the first rows because of missing values. * Two patients selected non-integer values in the range of 1–5 and their values were counted as missing.

### Patient Engagement Measures and SDM

We defined two patient engagement measures for use in analyzing the recorded encounters. We created the measures for this study to capture information unique to these treatment conversations. The measures were: 1) the number and type of questions patients asked; and 2) the number of times the patient expressed their preferences on treatment. The categories for type of questions appears in the Analytic Approach section. The preferences included stated preferences for any aspect of treatment, such as type of therapy and timing of treatment.

We assessed SDM using the OPTION12 Scale, which is a 12-item scale that rates clinician actions to promote SDM. The OPTION12 ranges from 0 (low SDM) to 100 (high SDM). The OPTION12 has been validated [18] and used for a variety of medical conditions including cardiovascular disease [19]. A prior study describes the methods for the collection of this measure [13]. Briefly, the OPTION12 was collected by two independent reviewers and concordance was assessed using the Lin concordance correlation coefficient. The concordance between the two reviewers was found to be high [13]. More information on the OPTION12 Scale components appears in Appendix Table 2.

**Table 2:** Regression Output for Knowledge Questions and Decisional Conflict Scale.

	Knowledge Coefficient (SE)	Decisional Conflict Coefficient (SE)

Adequate Health Literacy	–4.72 (4.92)	5.67 (2.88)*
Study Arm—Conversation aid	22.58 (4.48)***	–2.60 (2.76)
Constant	43.54	25.42

*** p<0.01, ** p<0.05, * p<0.1 *Note*: SE stands for standard error.

### Outcome Measures – Patient Knowledge and Decisional Conflict

We assessed outcome measures using the patient questionnaire data. The first outcome was patient knowledge about stable angina and the treatments. The assessment consisted of ten questions that were asked in the questionnaire immediately following the encounter. The questions related to information patients should understand after discussing treatment options for stable angina with their clinicians. These questions were developed for the original study with input from cardiologists who treat patients with stable angina [13]. We assessed the second outcome, decisional conflict, using the Decisional Conflict Scale (DCS). The DCS is a 16-item scale that measures patient perception of uncertainty and effective decision making. The scale score ranges from 0 (low conflict) to 100 (high conflict). The DCS has been validated [20] and applied to cardiovascular disease [21]. The specific questions we used to assess patient knowledge and the individual items for the DCS appear in Appendix Table 3.

### Analytic Approach

Two reviewers assessed the patient engagement measures we created for this study and extracted quotes from the recordings. To establish a consistent reviewing process, both reviewers first assessed the same ten recordings. We assessed the interrater reliability using the intraclass correlation coefficient (ICC) [22]. After assessing interrater reliability, the reviewers discussed discrepancies and divided the remaining recordings among themselves.

Initially, we divided the questions into the following categories: the risk of heart attack or death, the complications or side effects of treatment, symptom relief, clarification, and logistics. Logistics questions related to issues such as how long a coronary angiogram would take or whether a follow-up appointment was necessary. Clarification questions related to asking the clinician to explain information that had already been presented, but that the patient did not understand. However, after we conducted the interrater reliability evaluation we found that the ICCs were poor (0–0.4) for some of the categories because the two reviewers were having difficulty differentiating among some of the categories. To address the difficulty in classifying the questions, we grouped the question types into two larger categories that were more distinct: questions related to clinical aspects of treatment selection and questions related to logistics and clarification. The first category included the questions about the risk of heart attack or death, the complications or side effects of treatment, and symptom relief. After making this change to the categorization of questions, the ICCs were excellent (0.75–1) for the total number of questions asked in each category. The ICCs were also excellent (0.75–1) for the number of times patients expressed preferences.

We assessed the results for the patient engagement measures and SDM descriptively and compared the inadequate and adequate health literacy groups. We did not test the results for statistical significance because the recording sample was too small (53 patients, of which only 10 were classified as having inadequate health literacy). We illustrated key findings using extracted quotes.

We evaluated the results for the knowledge questions and decisional conflict by health literacy level. We assessed statistical significance using a Wilcoxon rank-sum test and ordinary least-squares regression while controlling for study arm.

### Sensitivity Analysis

We used an alternative specification for health literacy that incorporated two additional questions (see Appendix Table 1). We added the responses for all three questions together to form a scale from 0–12 points and defined patients with six or fewer points as having ‘inadequate’ health literacy [17]. In previous research, this alternative approach did not improve sensitivity or specificity [17]. Instead, the purpose of the sensitivity analysis was to assess whether the differences in patient knowledge and decisional conflict by health literacy level were dependent on which health literacy specification we used.

## Results

### Descriptive Statistics

Characteristics for the full sample and recording sample appear in Table 1. A majority of patients in both samples were male and most patients in the recording sample had a caregiver or family member attend with them. Patients with inadequate health literacy made up 19 percent (22/118) of the full sample and 19 percent (10/53) of the recording sample. Patients with inadequate health literacy were slightly older, more likely to only have a high school degree or less educational attainment, and had longer encounters than patients with adequate health literacy. Patients with inadequate health literacy were also more likely to receive PCI or CABG following the encounter. While half of patients (11/22) with inadequate health literacy received PCI or CABG, around a third of patients (31/96) with adequate health literacy received either procedure. Most patients in both the adequate (79/93) and inadequate (15/21) health literacy groups preferred to at least share in the decision-making process with their clinician. Only two patients (both in the inadequate health literacy group) preferred to defer decision making entirely to their clinician.

### Patient Engagement and Shared Decision Making

**Figure 1:**
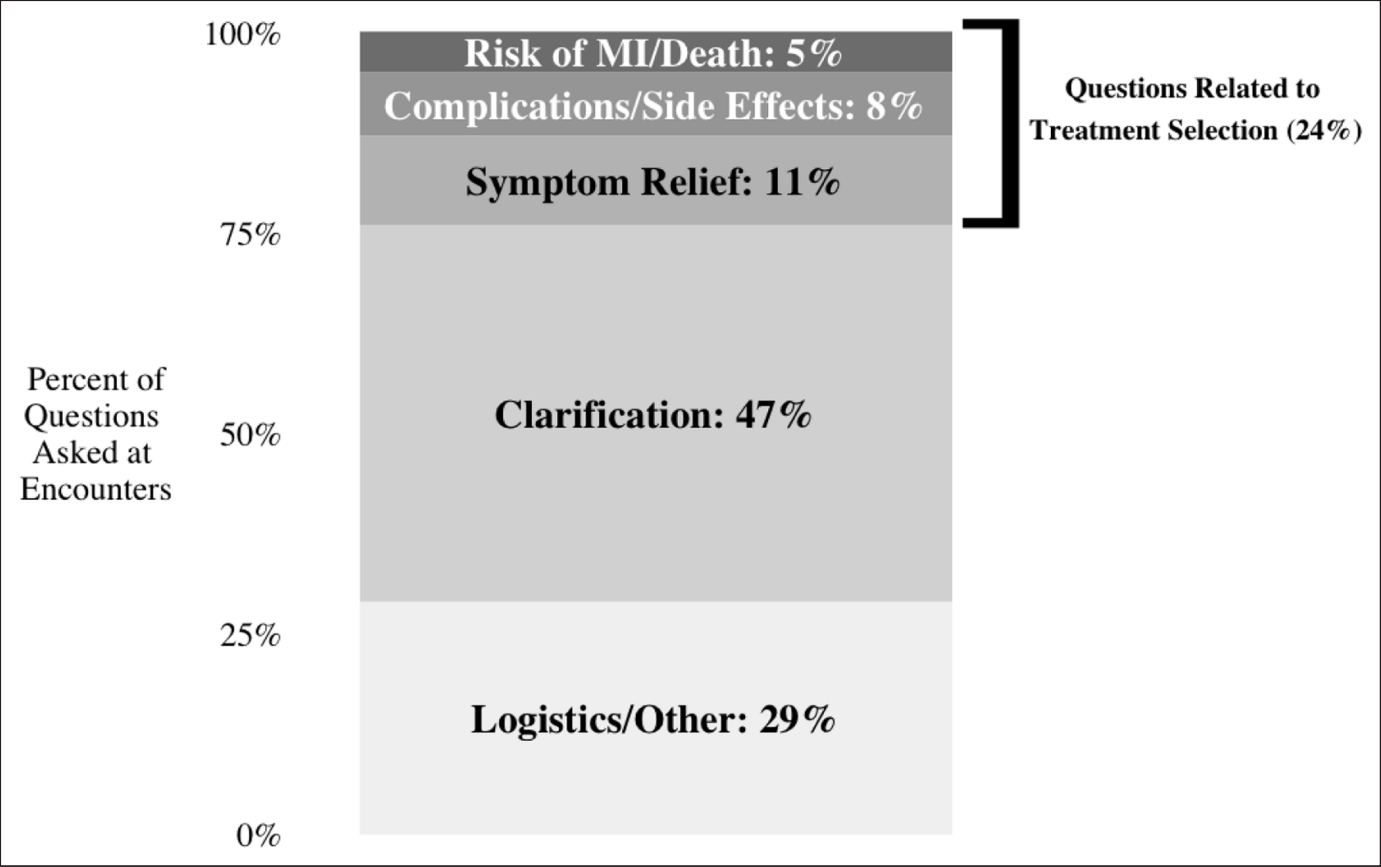
Topics of Patient Questions (N = 200) Asked at Encounters (N = 53). Note: ‘Clarification’ questions included questions that were related to the logistics of treatment, but that would not inform the decision between treatment alternatives. For example, one question in this category was about how the diagnostic angiogram would work. ‘Other’ questions related to information that was not directly relevant to the treatment of stable angina. Some questions included in the ‘Other’ category were about comorbid conditions or what is occurring in the heart.

Patients asked relatively few questions related to the clinical aspects treatment selection (Figure 1). Most questions related to the logistics of how the angiogram or PCI would work or clarification about information that was already presented.

An example of a question that relates to treatment selection is shown in **Box A**. This question asks about how patients experience symptom relief with PCI (stenting), which may affect the decision of whether to receive PCI. On average, patients asked 3.8 questions per encounter and only 24 percent (0.9 per encounter) of these questions were relevant to clinical aspects of treatment selection. About half of patients (27/53) asked no questions relevant to the clinical aspects of treatment selection, and a quarter asked only one such question (14/53). Patients with inadequate health literacy asked a similar number of questions related to clinical aspects (0.9 per encounter for both groups). However, patients with inadequate health literacy asked a greater number of questions about logistics and other issues (4.2 per encounter for inadequate health literacy vs. 2.6 for adequate health literacy patients).

**Box A**

Patient: “Do you feel better after you have a stent typically? Or is it just basically that you don’t notice anymore differences?”

Most patients expressed preferences for treatment, but often these preferences lacked a rationale or explanation. On average, patients expressed about two preferences per encounter (109 preferences and 53 encounters). Patients with inadequate health literacy expressed fewer preferences per conversation than patients with adequate health literacy (1.6 vs. 2.2). Also, 30 percent of patients with inadequate health literacy (3/10) expressed no preferences during the encounter compared to only 2.3 percent of patients with adequate health literacy (1/43). Over a third of patients did not express any preferences that included a rationale for the preference (19/53). A greater share of patients with inadequate health literacy did not express a preference with a rationale (6/10) than patients with adequate health literacy (13/43). In such instances, the patients would state their preferences without explaining the reason for their preference. In some of these cases, the patients would defer decision-making responsibility to their clinician, as in **Box B**, where the patient expresses a preference for the clinician to make a treatment recommendation.

**Box B**

Clinician: “I guess, you know, I will be guided by you. But if you felt that you really can’t make a decision, I am happy to suggest.”

Patient: “I was going to say, if you were me, what would you do at this juncture?”

In the recorded encounters, the reviewers observed that some clinicians appeared to have consistent patterns for how they engaged their patients across different encounters. One behavior that frequently arose was that some clinicians would ask patients leading questions, as in **Box C**:

**Box C**

Clinician: “So really, it’s all about how long you really wanna wait to have your symptoms relieved. Do you want immediate…or do you want…?”

Patient: “I want immediate.”

Clinician: “All right, that kind of tells me the answer then here.”

The clinician in this example appeared to be leading the patient to choose the option with faster symptom relief (PCI) by focusing on the advantage of PCI over medication only. Other clinicians seemed to ask questions in a more balanced way, as in **Box D**:

**Box D**

Clinician: “So based on that conversation, what do you think? I’m gonna take pictures today -and if I have a choice between medicines or stenting to make you feel better, which one do you think would be the right choice for you?”

**Figure 2:**
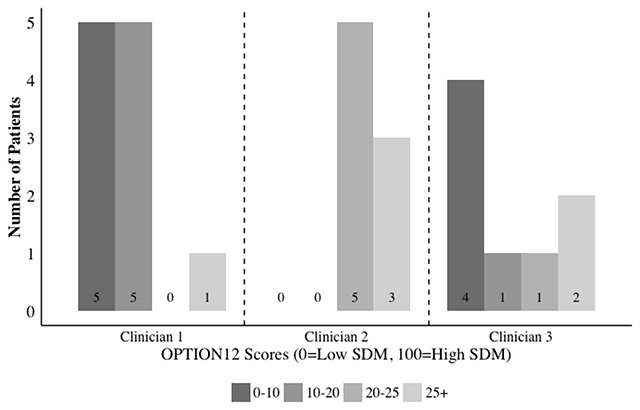
Shared Decision Making Scores for Clinicians with 8+ Encounters. Note: Shows OPTION12 Scores for Shared decision-making for the three clinicians in the study with eight or more patient encounters.

The SDM scores supported the observation that some clinicians having consistent patterns for engaging patients. The SDM scores for the three clinicians with at least eight encounters appear in Figure 2. Together, these clinicians accounted for half (27/54) of the conversations that were coded for SDM. The remaining half of the conversations involved 16 clinicians and each clinician had too few encounters to assess clinician patterns. Two of the three clinicians in Figure 2 (Clinicians 1 and 3) were cardiac catheterization nurses and one (Clinician 2) was an interventional cardiologist. Clinician 1 had consistently low SDM (OPTION12 scores below 25) for 10/11 patients. In contrast, Clinician 2 had high SDM (OPTION12 scores above 25) for 4/8 patients and had no scores below 20. While Clinician 1 and 2 had relatively consistent scores, Clinician 3 had six encounters with low SDM and two with high SDM. Clinician 3 also had a wide range in scores (4 for the lowest and 29 for the highest). However, the relative inconsistency for Clinician 3 may be explained by whether the conversation aid was used during the encounter. Clinician 3’s four lowest SDM scores came from usual care encounters.

Health literacy was not associated with SDM, but SDM itself was strongly linked to the outcome measures. The average SDM scores were slightly lower for patients with inadequate health literacy (18.5) than patients with adequate health literacy (19.5). A majority of patients in both health literacy categories (9/10 for inadequate and 31/43 for adequate health literacy) had encounters rated as low SDM. While the SDM scores did not have a strong association with health literacy, the scores did appear to be strongly associated with decisional conflict and patient knowledge. Patients who had high SDM encounters had lower decisional conflict (9.7 vs. 18.7) and higher knowledge scores (75 percent vs. 56 percent). However, most patients with high SDM (11/13) were also exposed to the conversation aid, so the differences may be due in part to study arm.

### Outcome Measures

**Figure 3:**
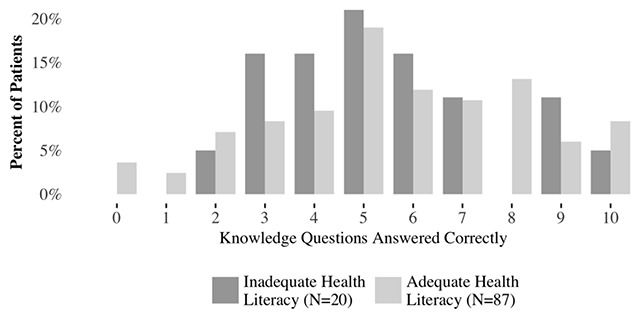
Distribution of Correct Responses to Knowledge Questions.

Patients showed a moderate but somewhat varied level of understanding in terms of their performance on the knowledge questions (Figure 3). On average, patients responded correctly to 55 percent of the questions. Patients performed slightly worse than average for the questions on whether PCI would reduce the risk of heart attack or death relative to medication only (51 percent) and whether patients who receive medication only have similar symptom relief compared to PCI at one year (46 percent). The mean and distribution of questions answered correctly were similar for patients by health literacy level (54 percent for inadequate health literacy and 56 percent for adequate health literacy) and the regression coefficient (Table 2) for inadequate health literacy was not statistically significant (p = 0.34).

**Figure 4:**
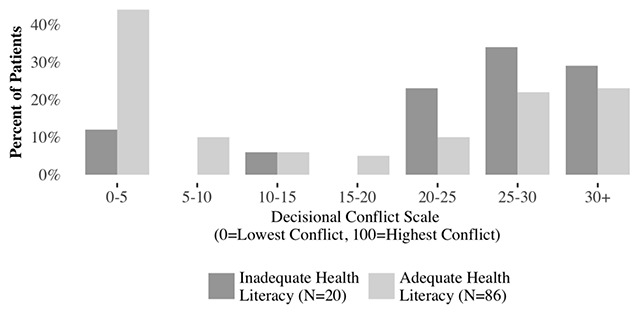
Distribution of Decisional Conflict.

Unlike the knowledge responses, health literacy was associated with decisional conflict (Figure 4). Average decisional conflict was greater among patients with inadequate health literacy (23.6 vs. 18.4). Similarly, the percentage of patients with decisional conflict greater than 25 (68 percent vs. 50 percent) was also higher in the inadequate health literacy group. A Wilcoxon rank-sum test for the association between health literacy level and decisional conflict was statistically significant (p = 0.019) and a regression coefficient (Table 2) for inadequate health literacy was not quite statistically significant (p = 0.051).

As noted above, the alternative specification for health literacy was used as a sensitivity analysis. The results were mostly consistent, although the rank-sum test became insignificant for decisional conflict (p = 0.18). However, the lack of statistical significance was partially due to fewer people being identified as having inadequate health literacy (11 vs. 19) using the alternative approach.

## Discussion

### Summary

This study provides a novel analysis of patient-clinician encounters to assess whether inadequate health literacy acts as a barrier to patient-clinician communication regarding treatment planning for stable angina. We found that patient engagement in terms of questions asked and SDM scores were similar for patients with inadequate and adequate health literacy. In contrast, patient with inadequate health literacy had fewer expressions of preference and were more likely to express no preferences during the encounter. Most patient questions in both groups related to logistical or clarifying information, and many patients asked no questions related to clinical aspects of treatment. However, patients were only able to answer about half of knowledge questions correctly. This finding suggests that many patients of all health literacy levels may lack a clear understanding of the treatment alternatives. While inadequate health literacy was not associated with less knowledge, patients with inadequate health literacy did have significantly higher decisional conflict than patients with adequate health literacy. We also found that a subset of three clinicians with the most encounters had consistent patterns in SDM. This result may mean that some clinicians have distinct communication styles that are consistent across encounters. Alternatively, this pattern may be explained by the type of clinician. The clinician with consistently high scores was an interventional cardiologist, whereas the clinicians with consistently low scores were catheterization nurses. However, this finding is exploratory since only three clinicians had sufficient observations to be analyzed.

### Prior Research

Several previous studies have analyzed patient-clinician discussions for other medical decisions [23,24,25]. One study examined the effectiveness of a decision aid for acute coronary syndrome among vulnerable sub-populations [25]. The study found that the decision aid improved trust in physicians to a greater extent among patients with low health literacy. However, this study did not find significant differences by health literacy level for the percentage of knowledge questions answered correctly, decisional conflict, and SDM. While the findings in this previous study with respect to knowledge questions and SDM were similar to the current analysis, the non-significant finding with respect to decisional conflict was different. The discrepancy may be due to differences in the analytical approach or confounding that could not be controlled for in this study because of the small sample size [25]. Another study analyzed recorded patient-clinician interactions for rectal cancer treatment planning with an approach that was similar to the current study [23]. The authors found that patient values were expressed in fewer than half of the interactions and that patient treatment preferences were expressed in fewer than a quarter of interactions [23]. These findings were consistent with the current study’s findings of low overall patient involvement in treatment planning. A systematic review of SDM scores (as measured by the OPTION12) for a variety of conditions [19] found a mean SDM score (23) that was similar to the mean in this study (19.5) [19]. Previous studies have also examined the relationship of health literacy with treatment planning. But more consistent research is needed to understand how the results in this study relate to health literacy and decision making in general [26].

### Implications for Practice

The findings from this study suggest potential targets for intervention. We found that both patients with inadequate and adequate health literacy asked relatively few questions about the clinical aspects of treatment selection. Also, patients with inadequate health literacy may have greater decisional conflict and were less likely to express preferences for treatment. Prior research suggests that many patients actually prefer to defer health decision making to their clinicians rather than engage in shared decision making [27]. For such patients, it may make sense for there to be fewer questions about the clinical aspects of treatment selection and fewer expressions of preferences. However, in our sample we found that a majority of patients in both the adequate and inadequate health literacy groups preferred to at least share decision making responsibility with their clinicians. One potential way to address the lack of involvement among patients who prefer greater involvement would be to have more time available for patients to discuss treatments alternatives and express preferences with their clinicians. SDM scores are consistently higher when consultations are longer [19].

An intervention to encourage clinicians to involve patients more is a conversation aid. As noted, greater patient involvement was one of the key goals for the PCI Choice conversation aid. However, the results of the trial were somewhat disappointing. While patients in the treatment arm had significantly higher knowledge scores, these patients did not have significant improvements in SDM or decisional conflict [13]. Follow-up interviews found that clinicians were initially unfamiliar with SDM and uncomfortable changing their practice patterns [15]. In particular, many clinicians used the conversation aid as an education tool rather than as a guide to support the conversation with the patient [13,15]. This observation is consistent with the finding that two of the three clinicians with sufficient observation had consistent communication patterns and involvement of patients regardless of study arm. Therefore, conversation aids such as PCI Choice may be still be effective when paired with additional training for clinicians.

### Limitations

This analysis has several limitations. First, the sample size was small, and few patients were categorized as having inadequate health literacy (10/53 for the recording sample, 22/118 for the full sample). Given the small sample size and descriptive nature of the analysis, the findings are exploratory in nature. Second, the analysis did not include racial or ethnic minorities in the recording sample and only one minority patient in the full sample. An extensive literature indicates that patient-clinician communication may differ when the patients are racial or ethnic minorities [28,29]. Third, the analysis took place at the Mayo Clinic, which is a referral center. The results may be different for other health systems such as those that have different cultures or serve different patient populations. Fourth, the OPTION12 score may not fully reflect whether meaningful SDM between patients and clinicians actually occurred. It was observed that some clinicians would go through the steps of the conversation aid as if it were an educational tool and still have high SDM scores. Fifth, the health literacy measure was a screening question instead of an instrument such as the S-TOFHLA or REALM. While the screening question has been validated against these instruments, it is associated with greater error in categorizing patients [16,17]. A particular concern is that the response to the screening question may be associated with decisional conflict since they both relate to confidence. A sensitivity analysis that used two additional screening questions for health literacy found consistent results. Sixth, the data originated from an RCT that randomized patients to receive a conversation aid or usual care. While the RCT found that the conversation aid only led to significant improvements in patient knowledge [13], it may have affected other aspects of the encounters such as the number and type of questions asked. We did not control for the study arm in the descriptive analysis of the patient engagement measures due to the limited sample size. We separately assessed the association of the conversation aid with patient engagement measures and found only small differences between the arms. It is also possible that there was contamination across the arms because some clinicians treated patients in both arms. However, the original RCT found no evidence that contamination had occurred [13]. Seventh, the act of recording the conversations may have changed patient or clinician behavior in response to being recorded (the Hawthorne Effect [30]). For example, clinicians may have engaged in more SDM because they knew they were being recorded and evaluated.

### Lessons Learned

While conducting this analysis, we also developed several insights into how data can be leveraged for research that has implications for care delivery. The recorded patient-clinician encounters were a rich data source for examining the quality of patient-clinician interactions. Through these recordings, we were able to directly assess the level of patient engagement instead of relying on clinician or patient self-report following the encounter. As delivery systems increasingly seek to promote SDM and patient-centered care, recorded encounters are a valuable resource for assessing such efforts. The main drawback of the recorded encounters was that the sample size of patients and clinicians was relatively small, which made it difficult to analyze the sub-group of patients with inadequate health literacy and the communication patterns of individual clinicians. In general, it may be difficult to record and qualitatively evaluate larger samples of encounters due to time constraints and the reluctance of some patients and clinicians to be recorded. Another takeaway was the value of the health literacy screening questions. The analysis was dependent on the inclusion of this measure for health literacy from the original PCI Choice Trial. The PCI Choice Trial questionnaires included the health literacy screening questions because they only required adding three additional questions. It would not have been feasible to include lengthier individual assessments of health literacy due to resource and time constraints. The analysis demonstrates that the health literacy screening questions can be used for research into the relationship of health literacy and care delivery. Delivery systems could potentially incorporate these screening questions for particular patient populations to enable research into disparities in care delivery and outcomes by health literacy level and to assess the impact of interventions designed to narrow these disparities. The main drawbacks of this measure were that it used a binary cutoff for health literacy categories and most patients fell into the adequate health literacy category. The imbalance in the two groups made it difficult to draw conclusions about the association of health literacy with the patient engagement measures and the outcomes. This was especially the case for the recording sample in which only ten patients were in the inadequate health literacy category.

## Conclusions

This analysis provides key insights into how inadequate health literacy may affect patient-clinician communication during treatment planning discussions. The results also suggest possible targets for intervention including providing additional time during encounters and improvements to clinician communication such as fostering choice awareness for patients of all health literacy levels. Future research could confirm these exploratory findings for different and larger populations.

## Additional Files

The additional files for this article can be found as follows:

10.5334/egems.306.s1 Appendix Figure 1. Sample Flow Diagram.

10.5334/egems.306.s2 Appendix Table 1. Health Literacy Measure.

10.5334/egems.306.s3 Appendix Table 2. OPTION12 Questions.

10.5334/egems.306.s4 Appendix Table 3. Outcome Measures.
