# Supplementary material for: Patient-Clinician Decision Making for Stable Angina: The Role of Health Literacy
Source: EGEMS (Wash DC). 2019 Aug 9;7(1):42. doi: 10.5334/egems.306 (PMC6688543; doi:10.5334/egems.306)
Supplement: Appendix Figure 1. — Sample Flow Diagram. [file egems-7-1-306-s1.pdf]

Appendix Figure 1: Sample Flow Diagram

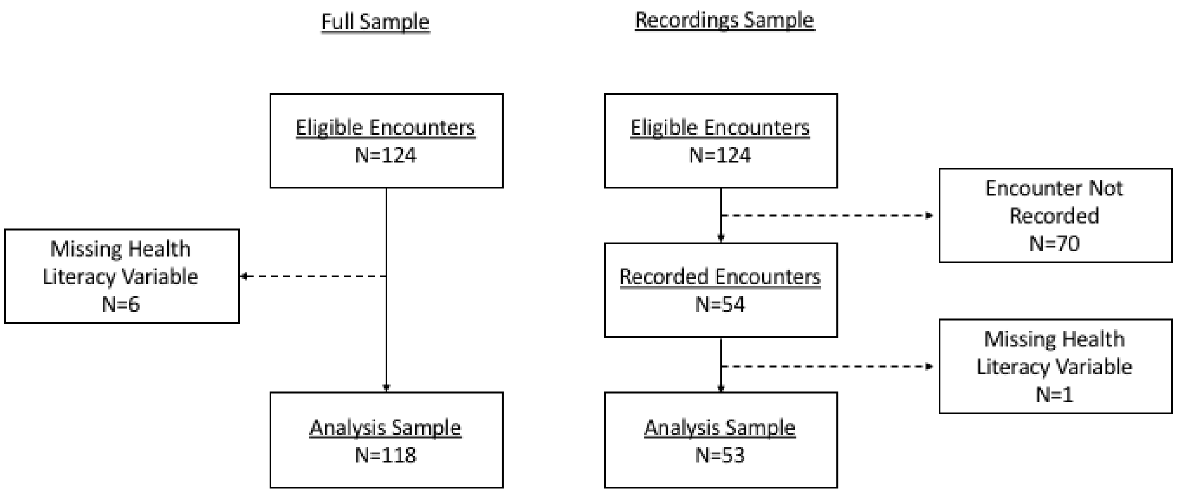

Note: The sample for some analyses is smaller due to missing data on outcome variables.
