# Supplementary material for: Patient-Clinician Decision Making for Stable Angina: The Role of Health Literacy
Source: EGEMS (Wash DC). 2019 Aug 9;7(1):42. doi: 10.5334/egems.306 (PMC6688543; doi:10.5334/egems.306)
Supplement: Appendix Table 1. — Health Literacy Measure. [file egems-7-1-306-s2.pdf]

Appendix Table 1: Health Literacy Measure

Measure	Definition (From Patient Pre Survey)
Health Literacy	**ITEMS 8:** How often do you have someone (like a family member, friend, hospital/clinic worker, or caregiver) help you read hospital materials?<br>1) All of the time<br>2) Most of the time<br>3) Some of the time<br>4) A little of the time<br>5) None of the Time<br>**ITEM 9:** How often do you have problems learning about your medical condition because of difficulty understanding written information? [Same responses as above]<br>**ITEM 10:** How confident are you filling out healthcare forms by yourself?<br>1) Extremely<br>2) Quite a bit<br>3) Somewhat<br>4) A little bit<br>5) Not at all
