# Supplementary material for: Patient-Clinician Decision Making for Stable Angina: The Role of Health Literacy
Source: EGEMS (Wash DC). 2019 Aug 9;7(1):42. doi: 10.5334/egems.306 (PMC6688543; doi:10.5334/egems.306)
Supplement: Appendix Table 2. — OPTION12 Questions. [file egems-7-1-306-s3.pdf]

Appendix Table 2: OPTION12 Questions

Measure	Definition
1. OPTION12 Scale	The OPTION12 is a twelve-item scale that measures the degree to which clinicians involve patients in medical decision-making. The items are scored 0-4 as follows: no effort (0), minimal effort (1), some effort (2), skilled effort (3), and exemplary effort (4). The items are:<br><br>1. The clinician draws attention to an identified problem as one that requires a decision-making process<br>2. The clinician states that there is more than one way to deal with the identified problem (‘equipoise’)<br>3. The clinician assesses the patient's preferred approach to receiving information to assist decision making<br>4. The clinician lists ‘options’, which can include the choice of ‘no action’<br>5. The clinician explains the pros and cons of options to the patient (taking ‘no action’ is an option)<br>6. The clinician explores the patient's expectations (or ideas) about how the problem(s) are to be managed<br>7. The clinician explores the patient's concerns (fears) about how problem(s) are to be managed<br>8. The clinician checks that the patient has understood the information<br>9. The clinician offers the patient explicit opportunities to ask questions during the decision-making process<br>10. The clinician elicits the patient's preferred level of involvement in decision making<br>11. The clinician indicates the need for a decision-making (or deferring) stage<br>12. The clinician indicates the need to review the decision (or deferment
