# Supplementary material for: Patient-Clinician Decision Making for Stable Angina: The Role of Health Literacy
Source: EGEMS (Wash DC). 2019 Aug 9;7(1):42. doi: 10.5334/egems.306 (PMC6688543; doi:10.5334/egems.306)
Supplement: Appendix Table 3. — Outcome Measures. [file egems-7-1-306-s4.pdf]

## Appendix Table 3: Outcome Measures

| Measure | Definition (From Patient Post Survey) |
|---|---|
| 1. Treatment Selection | **ITEM 1:** Which of the following best describes your current treatment plan for your stable coronary artery disease?<br>• Medicines alone<br>• Medicines plus stents<br>• Other, please specify |
| 2. Patient Knowledge | **ITEM 2:** Please respond to the following statements as best you can. [Responses: True, False, or Unsure]<br>a. Getting a stent for stable coronary artery disease will reduce my risk of heart attack or death when compared to medicines alone.<br>b. At 1 month, there is no symptom improvement with medicines alone.<br>c. At 1 month, more patients who got stents felt better when compared to patients on medicines alone<br>d. At 1 year, patients on medicines alone feel about the same when compared to patients who got stents<br>e. The added symptom relief with stents compared to medicines alone gets smaller over time<br>f. There is a risk of having a heart attack with the stent procedure itself<br>g. Patients getting a stent can suffer more bleeding than patients on medicines alone.<br>h. Stents eliminate symptoms of angina in all patients<br>i. In 100 people who initially choose medicines alone, more than half will go on to need a stent during the next year<br>j. In 100 people who initially choose a stent, 7 will need another procedure for stable coronary artery disease during the next year<br>The total score for this outcome is the number of questions answered correctly. |
| 3. Patient Perceived Understanding | **ITEM 2:** Thinking about the conversation that you had with your clinician today about stents plus medicines versus medicines alone for stabl coronary artery disease, please mark the response that best describes your agreement with the following statements.<br>[Responses Are: 1) Strongly Agree, 2) Agree, 3) Neither Agree Nor Disagree, 4) Disagree, and 5) Strongly Disagree]<br>a. I know which options are available to me<br>b. I know the benefits of each option<br>c. I know the risks and side effects of each<br>Each of the questions will be assessed independently since they relate to different aspects of treatment and the information for some aspects may be more difficult to understand than others. |

| | |
|---|---|
| 4. Patient Satisfaction | **ITEM 3:** Thinking about the conversation that you had with your clinician today about stents plus medicines versus medicines alone for stable coronary artery disease, please mark the response that best describes your agreement with the following statements.<br>[Responses Are: 1) Strongly Agree, 2) Agree, 3) Neither Agree Nor Disagree, 4) Disagree, and 5) Strongly Disagree]<br>a. I am clear about which benefits matter most to me<br>b. I am clear about which risks and side effects matter most to me<br>c. I have enough support from others to make a choice<br>d. I am choosing without pressure from others<br>e. I have enough advice to make a choice<br>f. I am clear about the best choice for me<br>g. I feel sure about what to choose<br>h. This decision is easy for me to make<br>i. I feel I have made an informed choice<br>j. My decision shows what is important to me<br>k. I expect to stick with my decision<br>l. I am satisfied with my decision<br>**ITEM 5:** How would you describe the clarity of information about stents plus medicines versus medicines alone for stable coronary artery disease given during this visit? [Responses Are: 1-7 from 1 “Too Little Information” to 7 “Too much Information”]<br>An overall patient satisfaction score will be created by summing the number of elements in Item 3 for which the patient responded “Strongly Agree” or “Agree.” Item 5 will be analyzed separately from the total patient satisfaction score. |
